# Development of a Freshness Indicator for Assessing the Quality of Packaged Pork Products during Refrigerated Storage

**DOI:** 10.3390/foods13132097

**Published:** 2024-07-01

**Authors:** Ga-Young Lee, Kyung-Jik Lim, Yoon-Hee Lee, Han-Seung Shin

**Affiliations:** Department of Food Science and Biotechnology, Dongguk University-Seoul, 32, Dongguk-ro, Ilsandong-gu, Goyang-si 10326, Republic of Korea; gatyoung99@naver.com (G.-Y.L.); kyung9209@naver.com (K.-J.L.); dldbsgml491@naver.com (Y.-H.L.)

**Keywords:** intelligent packaging, freshness indicator, total volatile basic nitrogen, food quality, pork

## Abstract

A pH-sensitive dye-based freshness indicator has been developed to monitor the quality status of pork neck through distinct color transitions, addressing a crucial need for improved food safety and real-time monitoring within the food industry. This system aims to boost consumer confidence and improve shelf-life estimates by offering transparent and immediate quality indicators. Aerobically packaged pork neck samples underwent accelerated testing at 25 °C for 36 h, followed by refrigeration experiments at typical distribution temperatures of 4 and 8 °C over 10 days. Measured pork neck quality parameters included total bacterial count (TBC), total volatile basic nitrogen (TVB-N), and pH levels. Visual observation and colorimetric analysis were used to assess the chromatic variations of the freshness indicator, which showed a significant shift from orange to green in response to the presence of TVB-N in the headspace of the pork packaging. The chromatic parameters of the freshness indicator exhibited a significant correlation with the pork quality values throughout the storage periods. The results highlight the ability of the freshness indicator to effectively convey quality information about pork through noticeable colorimetric changes.

## 1. Introduction

The growing interest is in natural products that are less processed and are better quality. Safety is a requirement for all food. Consumers demand accurate and transparent information about the sourcing, processing, and handling of food products throughout the supply chain. In response to these demands, food manufacturers, retailers, and regulators are implementing measures to increase transparency and provide consumers with access to reliable information. As a result, the concept of packaging that offers consumers additional functionality has become a key issue.

Food packaging serves as a marketing tool to communicate with consumers, protect the product from contamination by the external environment, and accommodate products of various sizes and shapes. Despite their crucial role, traditional packaging systems have inherent limitations. While isolating food from the external environment, traditional packaging systems lack the ability to convey information about the freshness and quality of packaged food to producers, retailers, and consumers [1]. Advances in packaging technology have led to the development of intelligent packaging systems in response to these challenges. Intelligent packaging refers to materials and articles that monitor the condition of packaged food or the environment surrounding the food [2]. This technology is growing in the food packaging sector because of its potential to improve food quality and safety, in addition to its convenience for consumers [3]. Intelligent packaging commonly integrates indicators to determine the condition, such as freshness, temperature, and gas [4,5]. Freshness indicators detect chemical changes within the product packaging to inform consumers of the quality status of food products. Generally, pH-sensing freshness indicators consist of a polymeric or biopolymeric base and a pH-sensing dye that reacts with chemicals produced in food during spoilage, such as basic nitrogen and organic acids [6]. These indicators offer real-time and convenient methods for monitoring the freshness of a product.

Throughout distribution and storage, microorganisms can grow, and cause physicochemical and biochemical changes that may instigate food degradation [7]. Meat, including pork, is a perishable product due to its high water activity and protein content. Pork typically exhibits a water activity of around 0.98 and contains approximately 21 g of protein per 100 g [8,9]. This makes it a prime environment for microbial growth and endogenous enzyme activity, leading to undesirable changes in color, odor, and texture [10,11]. Decomposed packaged meat can lead to food safety issues, such as food poisoning, and contribute to food waste. Therefore, it is essential to monitor meat quality throughout the supply chain, including manufacturing facilities, packaging facilities, transportation, and retail stores [12].

Traditional methods for assessing meat freshness include sensory evaluation and physical, chemical, and microbiological analysis [13]. However, these methods are time-consuming and laborious processes, making them unsuitable for real-time monitoring of meat product quality by consumers [14]. Freshness indicators can promptly discern the freshness status of packaged meat products through an immediately perceptible alteration. Therefore, they can contribute to the safety, quality, and convenience of meat products and prevent losses during distribution [15]. However, the practical implementation of freshness indicator technology is presently constrained by several challenges. These include scaling up production, securing regulatory approval, fostering consumer acceptance, improving the understanding of the correlation between response quality and various product categories, and enhancing the stability of the indicators [16].

The aim of this study is to develop a clearly perceptible pH-sensitive indicator for the freshness of pork that can change color by detecting volatile basic nitrogen generated from pork. In the putrefaction process in pork, volatile basic nitrogen compounds, such as trimethylamine, dimethylamine, and ammonia, are generated. This results in an alkaline atmosphere in the headspace of the packaging [17], causing a color change of the pH-sensitive indicator. The sensor for the freshness indicator is designed to be sensitive to volatile amines and to operate effectively under actual refrigeration temperatures, ensuring its practical application in meat distribution scenarios. In order to determine the effectiveness of the freshness indicator in reflecting the spoilage state of pork, pork spoilage indices were monitored throughout the storage period, and their correlation with the color changes of the indicator was evaluated.

## 2. Materials and Methods

### 2.1. Chemicals and Materials

The pork neck was purchased from a local butcher shop in Goyang-si, Gyeonggi-do, Republic of Korea. The pork was from the Yorkshire × Landrace × Duroc (YLD) breed and had been slaughtered within 3 days prior to purchase. Potassium carbonate, bromocresol green (BCG), and methyl orange (MO) were obtained from Samchun Chemicals Co., Ltd. (Seoul, Republic of Korea). Cellulose acetate, HCl, 0.01N standard H_2_SO_4_ solution, and methyl red-methylene blue solution were acquired from Daejung Chemical Co., Ltd. (Siheung, Republic of Korea). Acetone and ethyl alcohol were supplied by Ducksan Pure Chemicals Co., Ltd. (Ansan, Republic of Korea). Glycerol was procured from Sigma-Aldrich Co., Ltd. (St. Louis, MO, USA). Filter papers and the polytetrafluoroethylene (PTFE) membrane were provided by Hyundai Micro Co., Ltd. (Seoul, Republic of Korea). NaOH solution (40%) and potassium carbonate were purchased from Junsei Chemical Co., Ltd. (Tokyo, Japan).

### 2.2. Preparation of a pH-Sensitive Indicator for Application in Pork Packaging

The freshness indicator was prepared by immersing the filter paper in the indicator solution. The indicator solution was obtained by completely dissolving 3 g of cellulose acetate in 30 mL of acetone. Then, 1.5 g of glycerol was added. The optimum ratio of dye solution (20 mL) was added to the above cellulose acetate solution, and 100 µL of 1 M HCl was added to adjust the initial pH of the dye solution [18]. The dye solution was prepared by individually dissolving MO and BCG in 70% ethanol at a ratio of 0.1% (*w*/*v*). The filter paper was submerged in the prepared indicator solution for 10 min and dried at room temperature (20 °C) for 3 h. The indicator sensor was then cut into 2 cm × 2 cm pieces and placed on a hydrophobic gas-permeable membrane filter (PTFE-D), followed by lamination with a layer of polyethylene terephthalate (PET) film.

**Figure 1:**
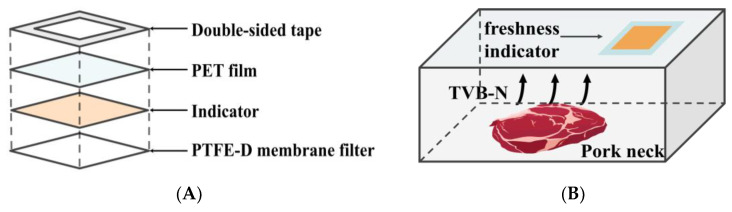
Schematic diagram illustrating the construction of the freshness indicator (**A**); Application of the freshness indicator for pork neck packaging (**B**).

The freshness indicator was designed to be attached to the inner surface of the package film, thereby exposing it to the headspace within the package. Figure 1 illustrates the structure and application of the prepared freshness indicator.

### 2.3. Assessment of Factors Contributing to Pork Spoilage

Polypropylene (PP) containers were used to contain 100 g of fresh pork neck samples, which were thermally sealed with polyethylene (PE) film and stored at the specified temperatures. The quality values of the pork neck were monitored at regular intervals to replicate acceleration tests and actual refrigerated conditions. These monitoring periods were conducted at room temperature (25 °C) and low temperatures (4 and 8 °C), with the frequency of monitoring being every 6 h at room temperature and every 2 days at low temperatures. The containers were prepared in accordance with the number of measurements conducted. Specifically, 6 containers were used at 4°C, 5 containers at 8°C, and 7 containers at 25 °C. Each container for each temperature condition underwent three repeated experiments with the pork neck samples.

#### 2.3.1. Measurement of Microbial Growth during the Decay of Pork

A 10 g sample of pork neck was mixed with 90 mL of sterile saline in a stomacher bag for the determination of the total bacterial count (TBC). The mixture was homogenized for 2 min using a stomacher (BagMixer^®^ 400 W, Interscience, Saint Nom La Breteche, France). Samples of serially diluted pork neck were then spread onto Petrifilm™ aerobic count plates (3 M, Elyria, OH, USA) and incubated at 35 °C for 48 h. Three plates were used for each dilution factor and incubated at 35 °C for 48 h. The number of colonies was counted and expressed as log CFU/mL.

#### 2.3.2. Determination of Total Volatile Basic Nitrogen Content in Pork

The microdiffusion method with a Conway unit was used to determine the total volatile basic nitrogen (TVB-N) content of pork neck [19]. Briefly, 5 g of pork neck sample was homogenized with 25 mL of distilled water using a homogenizer, and the homogenate was left for 30 min. Subsequently, the homogenates were centrifuged at 2000 rpm for 15 min and filtered through filter paper (Whatman No. 1). For the Conway dish test, 1 mL of 0.01 N H_2_SO_4_ was added to the inner section, and 1 mL of the sample along with 1 mL of potassium carbonate-saturated solution was added to the outer section. The Conway dish was then incubated at 25 °C for 1 h. After the addition of 10 µL of methyl red-methylene blue solution to the inner section, it was titrated with 0.01 N NaOH. The TVB-N content was calculated according to the following equation:

$$\mathrm{TVB}\text{-}N\left(\mathrm{mg}/100\;g\right)=0.14\;\times \;\left(b-a\right)/W\;\times \;100\;\times \;d$$

where a is the volume of 0.01 N NaOH used for sample titration (mL), b is the volume of 0.01 N NaOH added to the blank (mL), *W* is the sample weight (g), and d is the dilution factor.

#### 2.3.3. Analysis of pH Variation during the Pork Storage Period

The pork neck was minced, and 5 g of sample was homogenized in 20 mL of distilled water. The homogenate was then centrifuged at 2000 rpm for 15 min, and the supernatant was measured using a pH meter (Orion Star™ A211 Conductivity Benchtop Meter, Thermo Fisher Scientific, Waltham, MA, USA). The pH meter was calibrated using 4.01, 7.00, and 10.01 buffer solutions at room temperature (20 °C) prior to each measurement. All determinations were performed in triplicate.

### 2.4. Quantitative Evaluation of Colorimetric Changes in the Freshness Indicator

The color of the freshness indicator was evaluated using a portable colorimeter (CR-300, Minolta, Tokyo, Japan) and quantified using the CIE L*a*b* system. PP containers were used to contain 400 g of fresh pork neck samples. A freshness indicator was attached inside the PE film using double-sided tape, and the containers were thermally sealed with this film. Measurements were taken with the colorimeter positioned above the packaged film. Throughout each storage period, the freshness indicator was measured for L*, a*, and b* values in triplicate. In the CIE L*a*b* color space, L* represents brightness and ranges from 0 (black) to 100 (white), a* indicates redness (+) to greenness (−), and b* indicates yellowness (+) to blueness (−) [20]. The total color difference (Δ*E*) of the freshness indicator was calculated from the measured L*, a*, and b* values using the following equation:

$$\Delta E=\sqrt{{\left(L*-L_{0}*\right)}^{2}+{\left(a*-a_{0}*\right)}^{2}{+\left(b*-b_{0}*\right)}^{2}}$$

where *L*_0_*, *a*_0_*, and *b*_0_* are the initial L*, a*, and b* values of the freshness indicator.

### 2.5. Statistical Analysis

Statistical analysis was conducted using the IBM SPSS Statistics 23 software (IBM, Armonk, NY, USA). All experiments were performed in triplicate and expressed as the mean ± standard deviation (SD). A one-way analysis of variance (ANOVA) with Duncan’s post hoc test was performed to compare means at a significance level of *p* < 0.05. Correlations between the color changes of the freshness indicator and TBC, TVB-N, and pH were assessed using Pearson’s correlation coefficient (*r*).

## 3. Results and Discussion

### 3.1. Analysis of Chemical and Microbiological Indices during Pork Neck Degradation

Meat freshness is a complex concept that involves various factors, including microbiological, physicochemical, and biochemical attributes [7]. Identifying a single critical factor that definitively indicates the end of meat’s shelf-life poses a significant challenge. The degree of meat decay is typically evaluated through sensory testing, biological assays, and chemical analyses. Given that microbial growth is a primary cause of quality deterioration in most foods, the level of microbial proliferation correlates closely with sensory acceptability and serves as a marker for decay [21]. Furthermore, microbial activity results in the production of volatile amines and an increase in pH.

Therefore, this study conducted a comprehensive evaluation of TBC, TVB-N, and pH to determine the extent of pork spoilage.

#### 3.1.1. Bacterial Enumeration throughout the Spoilage Period of Pork Neck Samples

Assessment of microbial proliferation was conducted by measuring TBC. The growth of microorganisms causes meat spoilage and mainly contributes to a reduction in shelf-life. The high moisture content, nitrogen compounds, nutrients, and pH levels of meat promote rapid bacterial growth. Bacteria invading the surface or tissues of the meat cause abnormalities in its appearance, smell, and color, making it unsuitable for consumption. According to the Republic of Korea’s Ministry of Food and Drug Safety (MFDS), meat products are considered unacceptable if the TBC level exceeds 7 log CFU/g. Previous studies have shown that odor occurs when the surface TBC reaches approximately 7 log CFU/g in poultry stored under aerobic refrigerated conditions [22,23,24].

The changes in TBC of the pork neck samples at room temperature are given in Table 1. The TBC increased relatively steeply from 3.95 log to 5.65 log CFU/g during the first 6 h. Subsequently, it steadily increased and exceeded 7 log CFU/g after storage for 24 h at 25 °C. The findings indicate that storing pork shoulder samples at room temperature leads to rapid microbial growth. Zhao et al. reported that pork stored at 25 °C exhibited greater bacterial diversity compared to refrigerated conditions, thereby accelerating spoilage [25]. Despite a slower growth rate over time, bacterial proliferation persists, potentially causing TBC levels to surpass safe limits if stored for more than 24 h.

Table 2 and Table 3 indicate the increasing behavior of TBC at two low temperatures (8 and 4 °C). The initial TBC level was 4.06 log CFU/g under fresh conditions at 8 °C storage, escalating to 6.93 log CFU/g by day 4 (D4). During preservation at 4 °C, the TBC started at 3.90 log CFU/g, culminating at 6.85 log CFU/g on day 6 (D6), exhibiting slower microbial proliferation compared to the 8 °C condition. TBC exhibited a significant escalation throughout the storage duration, surpassing the critical threshold after day 8 (D8) at 4 °C and after day 6 at 8 °C, indicating a shelf-life disparity amounting to approximately 2 days. The pork microbial proliferation behavior observed in this study aligns with previous findings, which similarly observed microbial counts exceeding 7 log CFU/g on day 7 at refrigeration temperatures (4 °C and 5 °C) [26,27].

#### 3.1.2. Determination of TVB-N Contents in Pork Neck Samples

The increase in TVB-N compounds in meat is attributed to the microbial degradation of amino acids and the activity of endogenous enzymes. This process involves the conversion of precursors, such as L-carnitine and choline, in animal food into TVB-N [28]. Depending on the degree of spoilage, TVB-N can produce amines and hydroxide ions (OH^−^) in the presence of moisture [29]. Consequently, these compounds may react with the acidic pH dye in the freshness indicator, resulting in observable color changes. The Republic of Korea’s Ministry of Food and Drug Safety (MFDS) Notification (No. 2023-72) states that the standard value indicating initial spoilage based on the TVB-N content of fresh or frozen meat is 20 mg/100 g. Therefore, in this study, the shelf-life of pork neck was determined as the period during which the TVB-N content reached 20 mg/100 g.

As depicted in Table 1, a consistent escalation of TVB-N levels was observed with prolonged storage time at room temperature. It started at 6.3 mg/100 g and gradually rose to 25.2 mg/100 g after 36 h. There was a significant increase between 30 and 36 h, and the standard level was exceeded at 36 h. This finding is consistent with another study conducted by Zhang et al., which also reported that pork samples were not fresh after 36 h [30].

Table 2 and Table 3 illustrate the fluctuations in TVB-N content observed under refrigerated conditions (8 and 4 °C). With storage at 8 °C, the TVB-N level started at 6.30 mg/100 g and progressively escalated over time. The threshold value of 20 mg/100 g was exceeded between D4 and D6, indicating a degradation of meat quality. The initial TVB-N at 4 °C storage was 6.65 mg/100 g and exhibited a more gradual increase compared to the 8 °C storage. Between D6 and D8, there was a considerable increase in TVB-N, which reached 21.35 mg/100 g and exceeded the established threshold on D8. This trend was parallel to another study, which reported a TVB-N level of 19.07 mg/100 g on D6 during the storage of lean pork at 5 °C [26].

#### 3.1.3. Measurement of pH Changes during Pork Neck Storage

The breakdown of pork meat involves microbial and enzymatic activity that degrades proteins and results in the accumulation of volatile basic amines and other nitrogen compounds, with a tendency to increase pH, creating an alkaline environment [31]. According to the standards set by the MFDS, the pH spoilage criterion for meat products is 6.20–6.30.

Table 1 lists the changes in pH values of pork neck samples at room temperature. As indicated from the data, the initial pH value was 6.11, and a decrease to 6.03 was observed within the first 6 h. Over a period of 36 h, the pH value showed a consistent upward trend, starting at 6.07 at 6 h and gradually increasing to 6.45 at 36 h. The pH tended to decrease initially and then increase with subsequent storage periods. The early decrease in pH can be explained by glycogen degradation leading to lactic acid accumulation [32]. Subsequently, the increase in pH was correlated with the completion of rigor mortis, typically occurring 3–5 days after slaughter, and the increase in TVB-N content produced by microorganisms or endogenous enzymes [33,34]. Similar results for the initial decline in pH of beef samples have been reported by Lee et al. [35]. The pH value exceeded the standard criterion for the deterioration of freshness after 30 h of storage at 25 °C.

The pH values of the pork neck samples at two low temperatures are given in Table 2 and Table 3. Consistent with the 25 °C storage data, the pH at 4 °C showed an initial decline but increased progressively after day 2 (D2). Contrary to the findings at 4 °C, there was only a consistent upward trend at 8 °C storage. These differences can be attributed to the rapid degradation reaction of the pork neck at 8 °C, leading to an early decline in pH value before D2. The pH values were above the threshold level on D6 at 4 °C and on D4 at 8 °C. These findings align with earlier research that observed similar pH value trends [34].

### 3.2. Chromatic Measurement of the Freshness Indicator Using the CIE L*a*b* Color Space

Considering the above three factors (TBC, TVB-N, and pH), the shelf-life of pork neck was found to be approximately between 24 and 30 h when stored at 25 °C. Regarding the refrigeration temperatures, the shelf-life was found to be between D6 and D8 at 4 °C and between D4 and D6 at 8 °C. Similarly, Ran et al. observed a rise in TBC and TVB-N levels exceeding safety thresholds, rendering the pork unfit for consumption by D8 [36].

Volatile amines, having a higher pK_a_ than MO and BCG, are known to induce color changes in freshness indicators during the pork neck storage period [37]. The addition ratios of pH dye and the amount of HCl were adjusted to ensure that the freshness indicator reached its endpoint within the set shelf-life duration. Manipulation of the gas indicator sensitivity can be achieved by doping the indicator dyes with acid or alkaline substances [18]. The addition of HCl initially lowered the pH of the freshness indicator sensor, allowing the pH dye to maintain its acidic form, thereby controlling the reaction rate of the indicator. In this study, the appropriate ratio of MO and BCG dye solutions was set at 1:2, and 0.1 mL of 1N HCl was added. All freshness indicators were placed in pork neck packaging, and photographs were taken during storage until no further color changes were observed. The L*, a*, and b* values, as well as the total color differences (Δ*E*), were measured quantitatively using a colorimeter to assess the degree of color change in the indicators.

**Figure 2:**
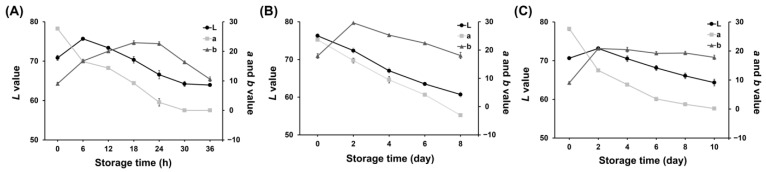
L*, a*, and b* values of the freshness indicator during storage at 25 °C (**A**); storage at 8 °C (**B**); storage at 4 °C (**C**).

In the 25 °C storage experiment of pork neck samples, there was a continual decline in the L* and a* values throughout the storage period, while the b* values exhibited a gradual increase followed by a subsequent decline after 24 h (Figure 2A). These numerical observations indicate a reduction in the brightness of the freshness indicator and a shift from a reddish to a greenish color.

Similar trends in the L*, a*, and b* values were observed during storage at two refrigeration temperatures. The L*, a*, and b* values measured in the freshness indicator of pork neck samples stored at 8 °C are illustrated in Figure 2B. Throughout the storage period, both the L* and a* values displayed a consistent decline. Initially, the b* value increased, but it started to decline after D2. Figure 2C shows the L*, a*, and b* values measured in the freshness indicator experiment conducted when pork neck samples were stored at 4 °C. The L* and a* values decreased over the storage time, with the a* value showing a noticeable change. This suggests a strong shift toward greenish hues throughout the period.

**Figure 3:**
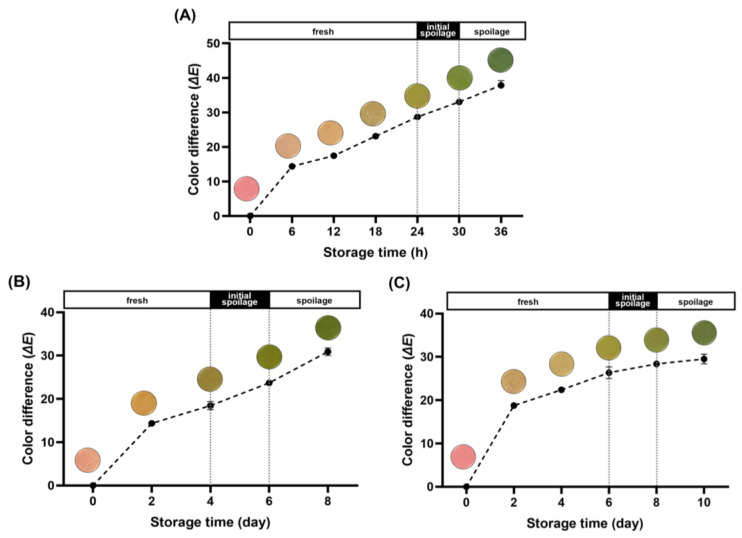
Color transitions and color differences (Δ*E*) of the freshness indicator during storage at 25 °C (**A**); storage at 8 °C (**B**); and storage at 4 °C (**C**).

The Δ*E* values, derived from the L*, a*, and b* values, quantify the numerical difference between two colors. They serve as a measure to represent the color variance between the initial state of the freshness indicator and its subsequent color changes. In general, the human eye can visibly perceive color variation at Δ*E* values above 5, which serves as an index to evaluate the color of the freshness indicator [38,39]. Figure 3A presents the Δ*E* values along with color changes observed in the acceleration experiment. The initial state of the freshness indicator was pink, transitioning to light orange by 12 h. Subsequently, it progressed to light green, ultimately reaching a complete green color between 24 and 30 h, aligning with the shelf-life duration established in the previous experiment. Correspondingly, the Δ*E* values progressively increased, reaching 28.71 and 33.06 during this interval. The color change of the freshness indicator depicted the phase of quality degradation in pork neck samples, providing an intuitive representation of the freshness level.

The optical changes and Δ*E* values of the freshness indicator at 8 °C are depicted in Figure 3B. Initially, it exhibited a pink color, gradually intensifying until reaching an Δ*E* value of 18.47 when it began transitioning to a green hue on D4. In Figure 3C, the color change and calculated Δ*E* values at 4 °C are illustrated. The alteration in color of the freshness indicator mirrored that observed at 8 °C, albeit at a slightly slower pace. Initially displaying a pink hue, the color transitioned to green from D6 onward and gradually deepened. The Δ*E* value steadily rose, reaching 26.34 at the end of the designated shelf-life and further increasing to 29.52 on day 10.

A tendency for the Δ*E* values of the freshness indicator to drop slightly with decreasing storage temperature was manifested when comparing the acceleration experiment with the actual refrigeration temperature experiments. This trend can be attributed to differences in microbial and enzymatic activity associated with temperature fluctuations. However, even at refrigeration temperatures, noticeable color and Δ*E* value changes were observed once spoilage had started. These results suggest that the freshness indicator developed in this study effectively represents the quality of pork neck, not only under accelerated experimental conditions but also under refrigerated temperature conditions.

### 3.3. Assessment of the Correlation between Chemical, Microbiological Parameters, and Color Changes of the Freshness Indicator

The correlation between the chemical and microbiological parameters (TBC, TVB-N, and pH) and the chromatic parameters (L*, a*, b*, and Δ*E* values) of the freshness indicator was examined to evaluate the effectiveness of the freshness indicator in reflecting the freshness status of pork neck. Table 4 lists the correlation coefficients (*r*) between the color variables of the freshness indicator and pork decay index during storage at 25 °C. The results revealed that the L* value exhibited the highest negative correlation with pH (*r* = −0.904, *p* < 0.01) and the lowest negative correlation with TBC (*r* = −0.754, *p* = 0.05). This low correlation between the L* value and TBC may be attributed to the rapid microbial growth in pork stored at relatively high temperatures. Moreover, the a* value indicated significant negative correlations with all three analyzed factors (*r* = −0.829 to −0.991, *p* < 0.05). In contrast, the b* value did not show significant correlations with any parameters, likely due to the color it represents having minimal relevance to the freshness indicator colors developed in this study.

The correlation coefficients (*r*) at refrigeration temperature are listed in Table 5 and Table 6. The L* value displayed significant negative correlations (*r* = −0.826 to −0.987, *p* < 0.05) with the pork spoilage indices at both 4 and 8 °C. The a* value presented strong negative correlations (*r* = −0.955 to −0.975, *p* < 0.05) with all three parameters at 8 °C. However, at 4 °C, the a* value showed a low correlation with pH, which is presumed to be due to the initial decrease rather than a consistent increase in pH during the storage experiments at this temperature.

Overall, both the L* and a* values were determined to have negative correlations with the chemical and microbiological parameters of pork neck. This inverse relationship indicates that as spoilage factors increase, there is a corresponding decrease in brightness and an increase in green coloration.

The Δ*E* values, which represent color differences discernible by the human eye, were evaluated for correlation with the freshness of pork neck at each temperature. A significant positive correlation was found (*r* = 0.76 to 0.99, *p* < 0.05) between Δ*E* values and pork spoilage parameters during storage of pork neck at 25 °C (Table 4). Moreover, strong positive correlations between Δ*E* values and both TBC and TVB-N were observed at both distribution temperatures, 4 and 8 °C (r = 0.84 to 0.97, *p* < 0.05), excluding the correlation between Δ*E* and pH at 4 °C (Table 5 and Table 6). In summary, the Δ*E* value of the freshness indicator effectively reflected the quality of pork in the order of TBC, TVB-N, and pH. The relatively weak correlation between the Δ*E* values of the freshness indicator and pH levels at 4 °C, as previously mentioned, may stem from the initial decline in pH due to lactic acid formation in the pork neck.

## 4. Conclusions

The freshness indicator developed in this study effectively signals the degradation of pork neck through colorimetric changes in response to increased levels of volatile amines. Starting in a pink state, the indicator transitions to green as it crosses the freshness threshold. The sensitivity and visibility of the freshness indicator are enhanced by the use of a combination of MO and BCG with the addition of HCl. The measured color shifts manifest a strong correlation with fluctuations in pork neck quality parameters under both room and refrigerated environments. The measured color transitions of the indicator consistently mirrored fluctuations in the quality parameters of pork neck, allowing for reliable real-time monitoring. The developed freshness indicator can be implemented in practical applications under refrigeration temperatures during distribution and storage processes, and its applicability can be extended to various types of meat and food products by modifying the quantity of additives. This study supports the potential of the freshness indicator to improve monitoring practices within livestock cold chain systems. This technology could enhance quality assessment processes and help reduce food waste. Additionally, it might be useful for stakeholders across the food supply chain, from producers to consumers, by offering a simple visual representation of food safety. However, there is enormous scope for further progress in freshness indicator technology. This study did not confirm the functionality of the freshness indicator under frozen conditions, so future research in this field should focus on experimenting with its effectiveness at freezing temperatures and improving its color stability. Additionally, addressing safety concerns is crucial, which can be achieved by preventing the migration of freshness indicators, dyes, or materials into food. By addressing these challenges, the technology can offer solutions to improving food safety and quality management across multiple food distribution and storage scenarios.

## Figures and Tables

**Table 1:** Alterations observed in TBC, TVB-N, and pH value of pork neck according to an accelerated experiment (25 °C).

	Storage Time (h)						
	0	6	12	18	24	30	36
TBC (log CFU/g)	3.95 ± 0.10 ^a^	5.65 ± 0.05 ^b^	6.12 ± 0.04 ^c^	6.90 ± 0.01 ^d^	7.45 ± 0.01 ^e^	8.19 ± 0.04 ^f^	8.54 ± 0.03 ^g^
TVB-N (mg/100 g)	6.30 ± 0.01 ^a^	9.10 ± 0.70 ^ab^	11.90 ± 0.70 ^bc^	14.35 ± 1.05 ^cd^	16.80 ± 0.01 ^de^	19.25 ± 0.35 ^e^	25.20 ± 2.80 ^f^
pH	6.11 ± 0.01 ^bc^	6.03 ± 0.01 ^a^	6.07 ± 0.01 ^b^	6.12 ± 0.01 ^c^	6.16 ± 0.02 ^d^	6.33 ± 0.01 ^e^	6.45 ± 0.01 ^f^

All values are the mean ± standard deviation of triplicate determination. Means with different superscripts in the same row are significantly different (*p* < 0.05) based on the Duncan test.

**Table 2:** TBC, TVB-N, and pH changes of pork neck under 8 °C storage conditions.

	Storage Time (Day)				
	0	2	4	6	8
TBC (log CFU/g)	4.06 ± 0.01 ^a^	5.87 ± 0.02 ^b^	6.93 ± 0.01 ^c^	8.35 ± 0.07 ^d^	8.59 ± 0.07 ^e^
TVB-N (mg/100 g)	6.30 ± 0.70 ^a^	8.05 ± 1.05 ^a^	16.80 ± 2.80 ^b^	22.75 ± 0.35 ^c^	24.50 ± 0.00 ^c^
pH	6.17 ± 0.02 ^a^	6.22 ± 0.02 ^b^	6.31 ± 0.01 ^c^	6.49 ± 0.01 ^d^	6.50 ± 0.02 ^d^

All values are the mean ± standard deviation of triplicate determination. Means with different superscripts in the same row are significantly different (*p* < 0.05) based on the Duncan test.

**Table 3:** TBC, TVB-N, and pH fluctuations of pork neck stored at 4 °C.

	Storage Time (Day)					
	0	2	4	6	8	10
TBC (log CFU/g)	3.90 ± 0.05 ^a^	4.75 ± 0.01 ^b^	6.18 ± 0.02 ^c^	6.85 ± 0.09 ^d^	7.03 ± 0.08 ^d^	7.50 ± 0.07 ^e^
TVB-N (mg/100 g)	6.35 ± 1.05 ^a^	8.85 ± 0.70 ^ab^	10.25 ± 1.05 ^b^	16.80 ± 2.80 ^c^	21.35 ± 0.35 ^d^	24.50 ± 0.80 ^e^
pH	6.11 ± 0.02 ^c^	5.90 ± 0.01 ^a^	6.03 ± 0.04 ^b^	6.35 ± 0.02 ^d^	6.53 ± 0.02 ^e^	6.61 ± 0.01 ^f^

All values are the mean ± standard deviation of triplicate determination. Means with different superscripts in the same row are significantly different (*p* < 0.05) based on the Duncan test.

**Table 4:** Correlation coefficient of variables related to pork spoilage with freshness indicator chromatic values at 25 °C.

	Parameter	L*	a*	b*	Δ*E*	TBC	TVB-N	pH
25 °C	L*	1	0.806 * p = 0.029	0.181 *p* = 0.698	−0.747 *p* = 0.054	−0.754 *p* = 0.050	−0.839 * p = 0.018	−0.904 ** p = 0.005
	a*	0.806 * p = 0.029	1	−0.130 *p* = 0.781	−0.993 ** p = 0.000	−0.991 ** p = 0.000	−0.975 ** p = 0.000	−0.829 * p = 0.021
	b*	0.181 *p* = 0.698	−0.130 *p* = 0.781	1	0.234 *p* = 0.613	0.248 *p* = 0.591	−0.003 *p* = 0.994	−0.406 *p* = 0.367
	Δ*E*	−0.747 *p* = 0.054	−0.993 *p* = 0.000	0.234 *p* = 0.613	1	0.998 ** p = 0.000	0.959 ** p = 0.001	0.764* p = 0.046
	TBC	−0.754 *p* = 0.050	−0.991 ** p = 0.000	0.248 *p* = 0.591	0.998 ** p = 0.000	1	0.953 ** p = 0.001	0.762 * p = 0.047
	TVB-N	−0.839 * p = 0.018	−0.975 ** p = 0.001	−0.003 *p* = 0.994	0.959 ** p = 0.001	0.953 ** p = 0.001	1	0.897 ** p = 0.006
	pH	−0.904 ** p = 0.005	−0.829 * p = 0.021	−0.406 *p* = 0.367	0.764 * p = 0.046	0.762 * p = 0.047	0.897 ** p = 0.006	1

* Statistically significant at *p* < 0.05; ** statistically significant at *p* < 0.01.

**Table 5:** Correlation coefficient between color values of the freshness indicator and pork TBC, TVB-N, and pH level of the pork neck at 8 °C.

	Parameter	L*	a*	b*	Δ*E*	TBC	TVB-N	pH
8 °C	L*	1	0.994 ** p = 0.001	0.181 *p* = 0.771	−0.969 ** p = 0.007	−0.986 ** p = 0.002	−0.986 ** p = 0.002	−0.968 ** p = 0.007
	a*	0.994 ** p = 0.001	1	0.190 *p* = 0.759	−0.979 ** p = 0.004	−0.975 ** p = 0.005	−0.967 ** p = 0.007	−0.955 * p = 0.011
	b*	0.181 *p* = 0.771	0.190 *p* = 0.759	1	0.003 *p* = 0.997	−0.046 *p* = 0.941	−0.290 *p* = 0.636	−0.290 *p* = 0.636
	Δ*E*	−0.969 ** p = 0.007	−0.979 ** p = 0.004	0.003 *p* = 0.997	1	0.978 ** p = 0.004	0.915 * p = 0.029	0.910 * p = 0.032
	TBC	−0.986 ** p = 0.002	−0.975 ** p = 0.005	−0.046 *p* = 0.941	0.978 ** p = 0.004	1	0.962 ** p = 0.009	0.961 ** p = 0.009
	TVB-N	−0.986 ** p = 0.002	−0.967 * p = 0.007	−0.290 *p* = 0.636	0.915 * p = 0.029	0.962 ** p = 0.009	1	0.982 ** p = 0.003
	pH	−0.968 ** p = 0.007	−0.955 * p = 0.011	−0.290 *p* = 0.636	0.910 * p = 0.032	0.961 ** p = 0.009	0.982 ** p = 0.003	1

* Statistically significant at *p* < 0.05; ** statistically significant at *p* < 0.01.

**Table 6:** Correlation coefficient between pork spoilage indices and color parameters of the freshness indicator at 4 °C.

	Parameter	L*	a*	b*	Δ*E*	TBC	TVB-N	pH
4 °C	L*	1	0.686 *p* = 0.132	−0.046 *p* = 0.931	−0.600 *p* = 0.208	−0.826 * p = 0.043	−0.935 ** p = 0.006	−0.987 ** p = 0.000
	a*	0.686 *p* = 0.132	1	−0.752 *p* = 0.084	−0.993 ** p = 0.000	−0.963 ** p = 0.002	−0.863 * p = 0.027	−0.666 *p* = 0.149
	b*	−0.046 *p* = 0.931	−0.752 *p* = 0.084	1	0.824 * p = 0.044	0.571 *p* = 0.237	0.350 *p* = 0.497	0.022 *p* = 0.967
	Δ*E*	−0.600 *p* = 0.208	−0.993 ** p = 0.000	0.824 * p = 0.044	1	0.926 ** p = 0.008	0.810 *p* = 0.051	0.578 *p* = 0.229
	TBC	−0.826 * p = 0.043	−0.973 ** p = 0.002	0.571 *p* = 0.237	0.926 ** p = 0.008	1	0.913 * p = 0.011	0.793 *p* = 0.060
	TVB-N	−0.935 ** p = 0.006	−0.863 * p = 0.027	0.350 *p* = 0.497	0.843 * p = 0.074	0.913 * p = 0.011	1	0.936 * p = 0.006
	pH	−0.987 ** p = 0.000	−0.666 *p* = 0.149	0.022 *p* = 0.967	0.578 *p* = 0.229	0.793 *p* = 0.060	0.936 ** p = 0.006	1

* Statistically significant at *p* < 0.05; ** statistically significant at *p* < 0.01.

## Data Availability

The original contributions presented in the study are included in the article, further inquiries can be directed to the corresponding authors.
